# Validation of plasma *p*‐tau217 as a Blood‐Based Biomarker for dementia diagnosis in Latin American population

**DOI:** 10.1002/alz70856_105295

**Published:** 2026-01-07

**Authors:** Ariel Caviedes, Felipe Cabral‐Miranda, Paulina Orellana, Carolina Gonzalez‐Silva, Hernan Hernandez, Andrea Slachevsky, Martin Alejandro Bruno, Hernando Santamaría‐García, Diana L Matallana, José Alberto Avila Funes, Francisco Lopera, María Isabel Behrens, Leonel Tadao Takada, Jennifer S. Yokoyama, Bruce L. Miller, Ken Kosik, Agustin Ibanez, Claudia Duran‐Aniotz

**Affiliations:** ^1^ Universidad Adolfo Ibañez, Santiago, Chile; ^2^ Latin American Brain Health Institute (BrainLat), Universidad Adolfo Ibañez, Santiago, Chile; ^3^ Federal Univesity of Rio de Janeiro, Rio de Janeiro, Rio de Janeiro, Brazil; ^4^ Latin American Institute for Brain Health (BrainLat), Universidad Adolfo Ibañez, Santiago, Santaigo, Chile; ^5^ Center for Social and Cognitive Neuroscience (CSCN), School of Psychology, Universidad Adolfo Ibañez, Santiago, Chile; ^6^ Universidad Adolfo Ibañez, Santiago, Santiago, Chile; ^7^ Neurology Service, Department of Medicine, Clínica Alemana, Universidad del Desarrollo, Santiago, Región Metropolitana de Santiago, Chile; ^8^ Memory and Neuropsychiatric Clinic (CMYN), Neurology Service, Hospital del Salvador and Faculty of Medicine, Universidad de Chile, Santiago, Chile; ^9^ Neuropsychology and Clinical Neuroscience Laboratory (LANNEC), Physiopathology Department ‐ ICBM, Neuroscience and East Neuroscience Departments, Faculty of Medicine, University of Chile, Santiago, Chile; ^10^ Geroscience Center for Brain Health and Metabolism (GERO), Santiago, Chile; ^11^ Instituto de Ciencias Biomédicas (ICBM) Facultad de Ciencias Médicas, Universidad Catoóica de Cuyo, San Juan, San Juan, Argentina; ^12^ Javeriana Univeristy, Bogota, Colombia; ^13^ Pontificia Universidad Javeriana, Bogota, Cundinamarca, Colombia; ^14^ Center for Memory and Cognition, Hospital Universitario San Ignacio Bogotá, Bogotá, Bogotá, Colombia; ^15^ Instituto Nacional de Ciencias Médicas y Nutrición Salvador Zubirán, México, DF, Mexico; ^16^ Grupo de Neurociencias de Antioquia, Facultad de Medicina, Universidad de Antioquia, Medellín, Antioquia, Colombia; ^17^ Departamento de Neurología y Neurocirugía, Hospital Clínico, Universidad de Chile., Santiago, Región Metropolitana de Santiago, Chile; ^18^ Clínica Alemana‐Universidad del Desarrollo, Santiago, Región Metropolitana de Santiago, Chile; ^19^ Centro de Investigación Clínica Avanzada (CICA), Universidad de Chile, Santiago, Región Metropolitana de Santiago, Chile; ^20^ Hospital das Clínicas, University of Sao Paulo Medical School, São Paulo, São Paulo, Brazil; ^21^ University of California, San Francisco, San Francisco, CA, USA; ^22^ Memory and Aging Center, Department of Neurology, Weill Institute for Neurosciences, University of California, San Francisco, San Francisco, CA, USA; ^23^ Global Brain Health Institute (GBHI), University of California San Francisco (UCSF); & Trinity College Dublin, San Francisco, CA, USA; ^24^ Global Brain Health Institute, University of California, San Francisco, San Francisco, CA, USA; ^25^ Memory and Aging Center, Weill Institute for Neurosciences, University of California, San Francisco, San Francisco, CA, USA; ^26^ University of California, Santa Barbara, Santa Barbara, CA, USA; ^27^ Global Brain Health Institute (GBHI), Trinity College Dublin (TCD), Dublin, Dublin, Ireland; ^28^ Global Brain Health Institute (GBHI), University of California San Francisco (UCSF); & Trinity College Dublin, Dublin, Ireland; ^29^ Latin American Institute for Brain Health (BrainLat), Universidad Adolfo Ibañez, Santiago, Chile

## Abstract

**Background:**

Plasma biomarkers have immense potential to transform dementia diagnosis globally, with phosphorylated tau 217 (*p*‐tau217) emerging as the most accurate AT(N) biomarker for diagnosis of Alzheimer's Disease (AD). Despite their promise, AT(N) biomarkers remain under‐validated in genetically and environmentally diverse populations, including Latin America (LA), where genetic, lifestyle, and social exposomes add layers of complexity. Diagnostic challenges like disease heterogeneity, limited research infrastructure, and restricted access to diagnostic tools further complicate diagnosis. In LA, research on plasma AT(N) biomarkers is scarce, and no studies in the global south have comprehensively integrated *p*‐tau217 with clinical or neuroanatomical correlates, leaving critical gaps in understanding its cognitive and brain‐specific associations. Leveraging the ReDLat cohort, comprising participants from six diverse LA countries (Chile, Argentina, Colombia, Peru, Mexico, and Brazil), this study aims to validate plasma *p*‐tau217 as a reliable biomarker for dementia diagnosis in AD and Frontotemporal Lobar Degeneration (FTLD). We hypothesize that *p*‐tau217, combined with cognitive assessments and neuroanatomical correlates, will serve as a robust diagnostic tool for dementia in LA populations.

**Method:**

A total of 600 participants (AD, FTLD, and healthy controls‐HC) were enrolled from the ReDLat cohort. Plasma samples were collected in EDTA tubes, aliquoted, shipped on dry ice, and stored at ‐80°C. Plasma *p*‐tau217 levels were quantified using the Lumipulse G600II system. Cognitive and neuroimaging data were used to evaluate associations with *p*‐tau217. Machine learning algorithms integrating plasma biomarker, cognitive, and neuroimaging data were developed to generate classification models.

**Result:**

*p*‐tau217 levels were significantly elevated in both AD and FTLD groups compared to HC. Plasma *p*‐tau217 demonstrated strong associations with cognitive domains, and its levels corresponded with structural and functional brain alterations. Machine learning models utilizing *p*‐tau217 achieved high diagnostic accuracy, with performance further improved by integrating neuroimaging and cognitive data.

**Conclusion:**

This multimodal study highlights the clinical utility of *p*‐tau217 as a blood‐based biomarker for dementia diagnosis in underrepresented LA populations. By addressing the region's genetic, environmental, and socioeconomic diversity, these findings establish a foundation for equitable diagnostic approaches and pave the way for broader implementation of plasma biomarkers in dementia diagnosis across diverse LA populations.

